# Lentiviral Vpx Accessory Factor Targets VprBP/DCAF1 Substrate Adaptor for Cullin 4 E3 Ubiquitin Ligase to Enable Macrophage Infection

**DOI:** 10.1371/journal.ppat.1000059

**Published:** 2008-05-09

**Authors:** Smita Srivastava, Selene K. Swanson, Nicolas Manel, Laurence Florens, Michael P. Washburn, Jacek Skowronski

**Affiliations:** 1 Cold Spring Harbor Laboratory, Cold Spring Harbor, New York, United States of America; 2 Stowers Institute for Medical Research, Kansas City, Missouri, United States of America; 3 Skirball Institute of Biomolecular Medicine, New York, New York, United States of America; University of Geneva, Switzerland

## Abstract

Vpx is a small virion-associated adaptor protein encoded by viruses of the HIV-2/SIVsm lineage of primate lentiviruses that enables these viruses to transduce monocyte-derived cells. This probably reflects the ability of Vpx to overcome an as yet uncharacterized block to an early event in the virus life cycle in these cells, but the underlying mechanism has remained elusive. Using biochemical and proteomic approaches, we have found that Vpx protein of the pathogenic SIVmac 239 strain associates with a ternary protein complex comprising DDB1 and VprBP subunits of Cullin 4–based E3 ubiquitin ligase, and DDA1, which has been implicated in the regulation of E3 catalytic activity, and that Vpx participates in the Cullin 4 E3 complex comprising VprBP. We further demonstrate that the ability of SIVmac as well as HIV-2 Vpx to interact with VprBP and its associated Cullin 4 complex is required for efficient reverse transcription of SIVmac RNA genome in primary macrophages. Strikingly, macrophages in which VprBP levels are depleted by RNA interference resist SIVmac infection. Thus, our observations reveal that Vpx interacts with both catalytic and regulatory components of the ubiquitin proteasome system and demonstrate that these interactions are critical for Vpx ability to enable efficient SIVmac replication in primary macrophages. Furthermore, they identify VprBP/DCAF1 substrate receptor for Cullin 4 E3 ubiquitin ligase and its associated protein complex as immediate downstream effector of Vpx for this function. Together, our findings suggest a model in which Vpx usurps VprBP-associated Cullin 4 ubiquitin ligase to enable efficient reverse transcription and thereby overcome a block to lentivirus replication in monocyte-derived cells, and thus provide novel insights into the underlying molecular mechanism.

## Author Summary

Monocyte-derived tissue macrophages play crucial roles in infection by primate lentiviruses. Human and simian lentiviruses of the HIV-2 and SIVsm/mac lineages encode a virion-bound virulence factor termed Vpx. Vpx is required to establish infection specifically of monocyte-derived cells, but the underlying molecular mechanism is unclear. In this study we characterize how the replication of SIVmac is blocked in the absence of Vpx and how Vpx overcomes this block. We find that Vpx is required for efficient reverse transcription of the incoming RNA genome, suggesting that Vpx acts early following virion entry into the macrophage, probably on events linked to virion uncoating and/or reverse transcription. We also identified a Vpx-associated ternary protein complex that is the key mediator of Vpx function specifically in macrophages. This complex links Vpx to the cellular machinery that mediates protein ubiquitination and degradation. Together, we describe the immediate downstream effector, the molecular machinery and a tentative mechanism that lentiviral Vpx uses to enable reverse transcription in macrophages. Our findings should lead to the conception of new strategies to control macrophage infection by human and simian lentiviruses.

## Introduction

Vpx accessory proteins are virulence factors encoded by viruses of the HIV-2/SIVsm/SIVmac lineage of primate lentiviruses. *vpx* gene disruption results in greatly reduced rates of virus replication in monocyte-derived cells, such as differentiated macrophages, but has no overt effect in primary T lymphocytes, as well as T and monocytic cell lines [1],[2],[3]. Intact *vpx* gene is required for optimal replication of these viruses in the infected host [4],[5]. Thus, it is thought that the role of Vpx in natural infection is to enable the establishment of virus reservoirs in macrophages.

Vpx is recruited into viral particles through the interaction with the p6 component of Gag [6],[7], and thus is available to facilitate an early event in the virus life cycle upon virion entry into the target cell. Indeed, an early study revealed that Vpx is required for efficient transport of preintegration complexes to the nuclei of infected macrophages [3]. In more recent studies HIV-2 and SIVsm Vpx proteins were found to promote accumulation of reverse transcribed viral genomes upon infection of dendritic cells (DCs) and this effect may reflect the ability of Vpx to overcome a proteasome dependent mechanism that inhibits an as of yet unidentified early event in the viral replication cycle [8]. How Vpx intersects this ubiquitin-dependent proteasomal protein degradation mechanism is unclear.

Vpx is a paralogue of Vpr accessory factor encoded by all known lineages of primate lentiviruses [9]. Although their amino acid sequences are closely related, the two proteins have different roles along the viral life cycle. For example, Vpr has the ability to activate DNA damage checkpoint and thereby arrest cells in the G2 phase of the cell cycle, while Vpx does not possess this function (reviewed in [10]). Results from recent proteomic studies revealed that lentiviral Vpr proteins associate with components of the ubiquitin proteasome system (UPS), such as Vpr Binding Protein (VprBP, GenBank NM014703) termed also DDB1 and CUL4-associated factor 1 (DCAF1), damaged DNA-binding protein 1 (DDB1, GenBank U18299), DET1 and DDB1 associated 1 (DDA1, GenBank DQ090952) and Cullin 4 (GenBank NM001008895, NM003588) ([11],[12],[13] reviewed in [14]). Cullin 4 is a scaffold protein that assembles a family of E3 ubiquitin ligase complexes. DDB1 is an obligatory subunit of all Cullin 4 E3's that bridges the catalytic cores organized on the Cullin 4 scaffold to a substrate-recruiting subunit, and VprBP/DCAF1 is a putative substrate adaptor for Cullin 4-based E3 ubiquitin ligases ([15] reviewed in [16],[17]). Evidence has been obtained showing that these interactions provide Vpr with the ability to modulate specifically the intrinsic catalytic activity of the Cullin 4 E3 containing VprBP and with a potential to influence the recruitment of substrate proteins for ubiquitination by Cullin 4, which in turn leads to the activation of DNA damage checkpoint [13],[18].

Since Vpx, similarly to Vpr, probably functions as an adaptor protein, we have used a combination of biochemical and proteomic methods to identify downstream effectors of Vpx encoded by the pathogenic SIVmac 239 strain. Here we show that SIVmac Vpx also binds DDA1-DDB1-VprBP complex, which links Vpx to Cullin 4, thus extending the previous observation that another SIV Vpx variant can bind VprBP [11]. Importantly, we demonstrate that VprBP, and its interaction with Vpx, are required for efficient macrophage transduction by SIVmac. Surprisingly, in the absence of Vpx, the incoming RNA genome is reverse transcribed very inefficiently. These findings indicate that Vpx facilitates macrophage infection by acting prior to and/or during reverse transcription, rather than by facilitating nuclear transport of the fully reverse transcribed preintegration complex, as has been thought previously ([3], reviewed in [10]). Together, our findings identify the UPS system and the VprBP associated protein complex as cellular machinery and immediate downstream effector that Vpx uses to promote replication of cognate primate lentiviruses in cells of monocyte/macrophage lineage, and provide novel insights into the underlying mechanisms.

## Results

### Vpx binds DDA1, DDB1 and VprBP components of the UPS

Two complementary strategies were used to identify cellular proteins that are bound by SIVmac 239 Vpx. As one approach, U937 monocytic cell populations were transduced with BABE-puro retroviral vectors stably expressing Vpx tagged at its N-terminus with a triple HA-FLAG-AU1 epitope tag (hfa-Vpx). The population of positively transduced cells was then selected with puromycin and expanded in spinner cultures for biochemical experiments. Surprisingly, we observed that U937 cells that stably expressed Vpx grew more slowly than the control U937 population transduced with an empty BABE-puro vector (data not shown), suggesting that Vpx is toxic and/or cytostatic to these cells. This in turn raised the possibility that chronic Vpx expression could lead to selection of escape variants where Vpx interaction with cellular proteins is not faithfully reproduced. Therefore, as an additional approach hfa-Vpx was expressed transiently in human embryonic kidney 293T (HEK 293T) cells by calcium phosphate co-precipitation. Next, Vpx and its associated proteins were purified from U937 and HEK 293T detergent extracts by sequential immunoprecipitations with anti-HA- and anti-FLAG- epitope antibodies, each followed by elution with the respective peptide epitope.

The immunoprecipitates were proteolyzed without prior separation of protein bands by SDS-PAGE and peptide mixtures analyzed by multidimensional protein identification technology (MudPIT, [19]). Interestingly, the most abundant cellular polypeptides we found associated with Vpx both in U937 and HEK 293T cells, but were absent from control purifications from cells that did not express Vpx were DDA1, DDB1 and VprBP/DCAF1 (see Table 1). Significantly, DDB1, an obligatory subunit of all known Cullin 4 based E3 ubiquitin ligases [16], VprBP, a known Vpr-binding cellular protein that has been recently shown to bind DDB1 and postulated to function as a substrate receptor for Cullin 4 E3 ubiquitin ligase [15],[20] and DDA1, a DDB1-binding protein that links to a negative regulator of Cullin4 E3 ubiquitin ligases [21], were thus identified as relatively abundant Vpx-associated proteins. Notably, DDB1, VprBP and DDA1 were recently shown to assemble a ternary complex that associates with Vpr proteins of HIV-1 and SIVmac and mediates activation of DNA damage checkpoint by these accessory factors [13]. Thus, our observations suggested that Vpx and its Vpr paralog both act through the DDA1-DDB1-VprBP complex, even though the two proteins execute distinct functions.

**Table 1:** MudPIT identification of cellular proteins that specifically associate with SIVmac Vpx in U937 monocytes and HEK 293T cells.

Cell line:	U937		HEK 293T	
Epitope tagged subunit:	SIVmac Vpx	Mock	SIVmac Vpx	Mock
Vpx	17^a^: **32.7** b		398; **76.0**	
**Ubiquitous:**				
DDB1	19; **10.7**		12; **6.6**	
VprBP	22; **11.2**		33; **16.8**	
DDA1	8; **38.2**		4; **26.5**	

(a) Spectral count, number of tandem mass spectrometry spectra matching peptides from the indicated protein.

(b) Sequence coverage, percentage of protein sequence represented in peptides identified by mass spectrometry.

**Figure 1:**
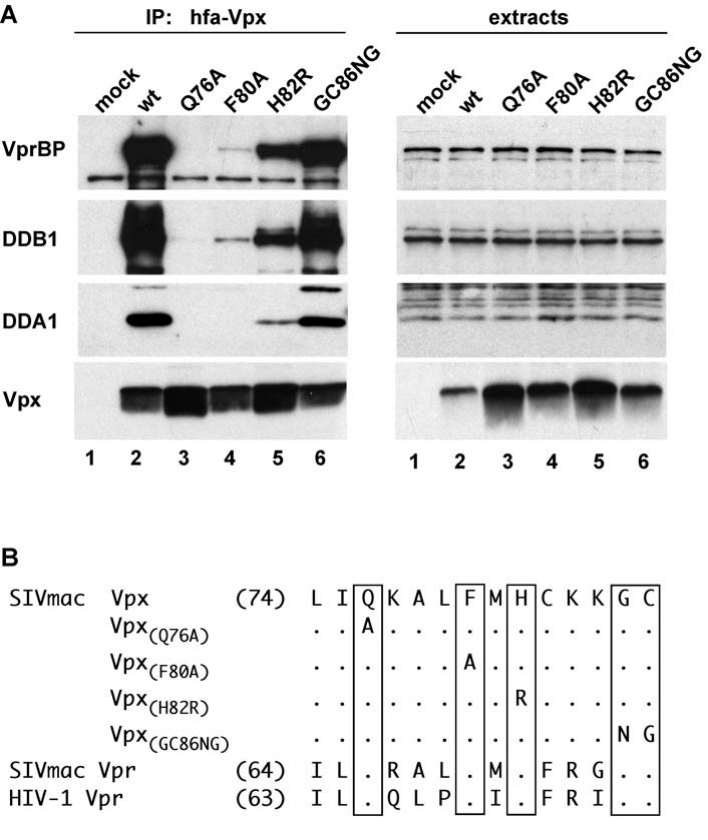
Conserved amino acid residues in Vpx C-terminal region mediate the association with DDA1-DDB1-VprBP complex. (A) hfa-tagged wild type (lane 2) or mutant (lanes 3–6) Vpx proteins were transiently expressed in HEK 293T cells and precipitated from detergent extracts with FLAG-M2 affinity resin. DDB1, VprBP, DDA1 and hfa-Vpx were detected in immune complexes (left panel) and cell extracts (right panel) by immunoblotting and visualized by enhanced chemiluminescence. (B) Amino acid sequences of the C-terminal regions of SIVmac 239 Vpx, SIVmac 239 Vpr, and HIV-1 NL43 Vpr are aligned and amino acid substitutions for the conserved residues in Vpx are indicated. Numbers indicate the positions of the first amino acid residue shown in each protein sequence. Dots identify amino acid identities and letters specify amino acids in the single-letter code.

To verify the data from MudPIT analyses, experiments were performed to confirm that Vpx associates specifically with the endogenous DDB1-VprBP-DDA1 complex. hfa-Vpx was transiently expressed in HEK 293T cells. Then, hfa-Vpx and its associated proteins were immunoprecipitated from detergent extracts prepared from the transfected cells with anti-FLAG-affinity resin, separated by SDS-PAGE and analyzed by western blotting with antibodies specific for VprBP, DDB1 and DDA1. As shown in Figure 1A, VprBP, DDB1 and DDA1 were readily detected in immune complexes isolated from hfa-Vpx expressing, but not from control, HEK 293T cells. Thus these data confirm that Vpx associates with DDB1, VprBP and DDA1 (compare lane 2 with 1).

### The C-terminal region of Vpx mediates binding to DDA1-DDB1-VprBP complex

The finding that Vpx associates with DDA1, VprBP and DDB1 was not entirely surprising because SIVmac Vpx amino acid sequence is approximately 25% and 50% identical to those of HIV-1 and SIVmac Vpr proteins, respectively, and because some of the previously tested SIVmac/HIV-2 Vpx variants were reported to bind VprBP [11],[22]. Previous studies have demonstrated that Vpr binds DDA1-DDB1-VprBP complex via its C-terminal α-helical region [11],[13]. Given the high degree of sequence identity between Vpx and Vpr proteins, this raised the possibility that Vpx binds the above complex in a manner similar to that seen with Vpr [11]. To test this and to develop mutant Vpx proteins defective for the interaction with DDA1-DDB1-VprBP, we substituted amino acid residues located in the C-terminal α-helical region of Vpx that are conserved in Vpr proteins (see Figure 1B). Mutant Vpx proteins were then transiently expressed in HEK 293T cells, immunoprecipitated via their FLAG tags, and immune complexes analyzed by Western blotting. As shown in Figure 1A, alanine substitution for the conserved glutamine Q76 (Q76A) disrupted Vpx ability to associate with DDA1, VprBP and DDB1. Also, alanine substitution for the conserved phenylalanine F80 (F80A) and arginine substitution for histidine 82 (H82R) had similar effects. Of note, the corresponding mutations in HIV-1 Vpr were previously shown to disrupt the binding to the VprBP-associated protein complex ([11], data not shown). Finally, mutating the conserved glycine G86 and cysteine C87 residues (GC86NG) did not have a detectable effect. We conclude that Vpx binds the DDA1-DDB1-VprBP complex via its C-terminal domain, probably using an interaction surface that is also conserved in the Vpr protein.

### VprBP links Vpx to the Cullin 4 scaffold

**Figure 2:**
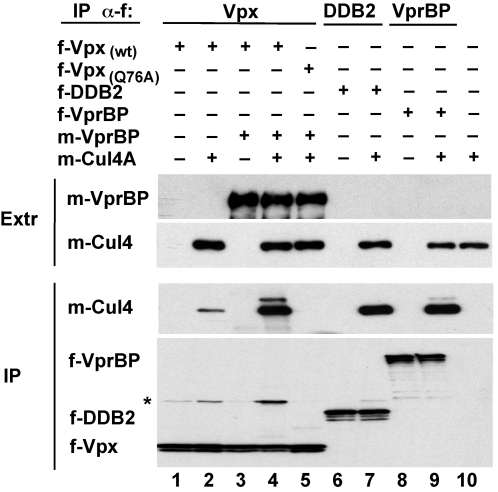
VprBP links Vpx to Cullin 4. myc-tagged Cullin 4A (m-Cul4) was coexpressed with FLAG- (f-) or myc- (m-) tagged Vpx, VprBP, DDB2 in HEK 293T cells, as indicated. Protein complexes were immunoprecipitated via their FLAG-tagged f-Vpx, f-VprBP, or f-DDB2 subunit, and resolved by SDS-PAGE. f- and m-tagged polypeptides were detected in detergent extracts (extr) and immune precipitates (IP) by immunoblotting with anti-FLAG or anti-myc epitope antibodies, respectively. Asterisk (*) indicated a background band that corresponds to the heavy chain of the anti-FLAG IgG.

The VprBP-DDB1 module was found to bind Cullin 4 and to participate in a functional Cul4-DDB1[VprBP] E3 ubiquitin ligase complex [13]. Therefore, we tested whether Vpx can associate with Cullin 4 and, if so, whether VprBP mediates this association. hfa-Vpx was transiently expressed together with myc-tagged Cullin 4A isoform (m-Cul4) and/or myc-tagged VprBP (m-VprBP) in HEK 293T cells, and anti-FLAG immunoprecipitates were analyzed for Cullin 4 by immunoblotting. As shown in Figure 2, VprBP co-expression dramatically elevated the levels of Cullin 4 associated with wild type Vpx, but not with the Vpx_(Q76A)_ variant that is unable to interact with VprBP and DDB1 subunits of the E3 complex (compare lane 4 with 2 and 5). We conclude that VprBP links Vpx to the Cullin 4-based E3 complex.

Notably, we observed that the Vpx-associated Cullin 4 migrated as a doublet (see lane 4). The slower migrating form of Cullin 4 was much less abundant in immune complexes assembled with VprBP in the absence of Vpx (lane 9), and co-migrated with the neddylated form of Cullin 4 shown previously to be induced by HIV-1 Vpr (see Figure S1, and ref. [13]). As expected, the upshifted Cullin 4 isoform was not detected in the E3 complex containing the DDB2 substrate receptor, which is catalytically repressed in the absence of damaged DNA (lane 7, see ref. [23]). Notably, in contrast to Vpr, the Vpx-induced modification was much less pronounced, and did not lead to a robust increase in catalytic activity of the associated E3 (Figure S1). We conclude that SIVmac Vpx is a much less potent inducer of Cullin 4 neddylation and E3 catalytic activity, than HIV-1 NL43 Vpr.

### Disruption of Vpx binding to VprBP compromises macrophage transduction by SIVmac

The ability of Vpx to enable infection of primary macrophages is well documented, yet the immediate downstream mediator(s) of Vpx remains unknown [1],[2],[3]. Therefore, experiments were performed to assess whether the interaction with VprBP and its associated E3 complex is important for Vpx's ability to facilitate macrophage transduction by SIVmac 239. Since this function is probably mediated by the virion-bound Vpx molecules, our initial experiments assessed the ability of the mutant Vpx proteins to be incorporated in SIVmac 239 virions.

**Figure 3:**
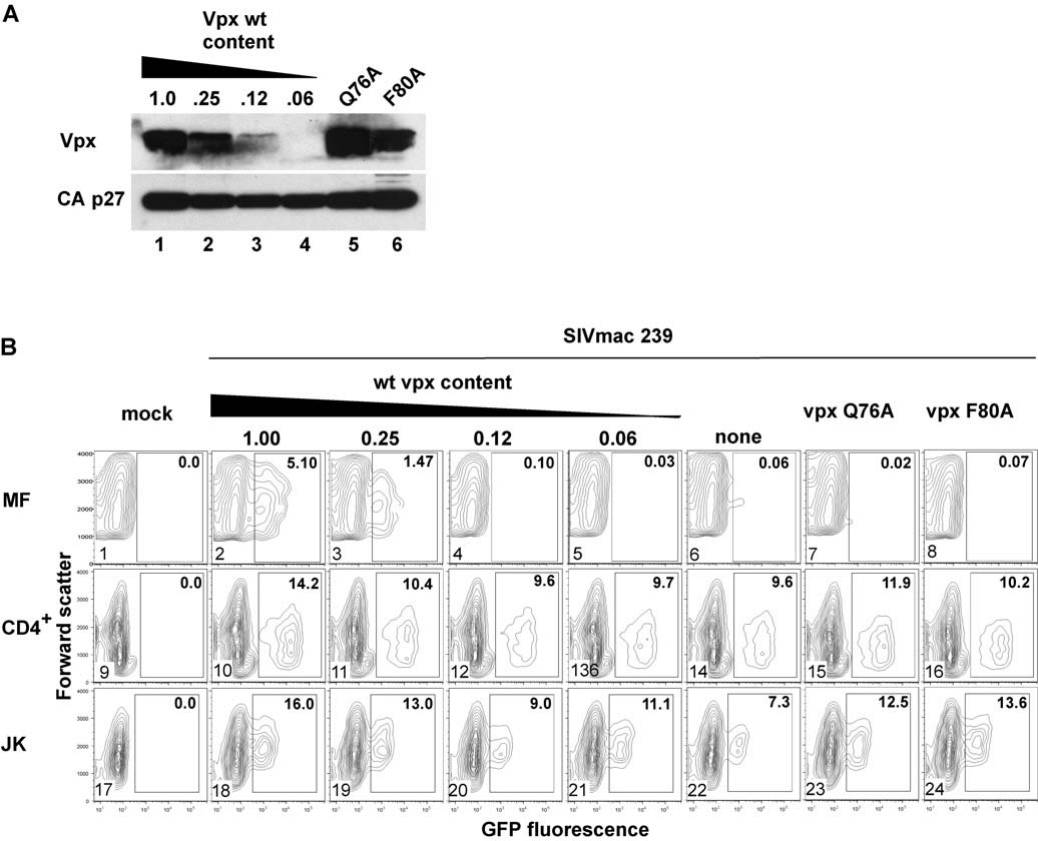
Q76A and F80A substitutions in Vpx disrupt ability of SIVmac 239 to transduce macrophages. (A) Q76A and F80A substituted Vpx are efficiently incorporated into SIVmac virions. Reference VSV-G pseudotyped single cycle SIVmac 239_(GFP)_ virions containing decreasing amounts of wild type Vpx were produced from HEK 293T cells transiently transfected with the SIVmac 239_(GFP)_ proviral clone containing wild type *vpx* gene alone (lane 1), or co-transfected with isogenic proviral clones containing wild type or inactivated *vpx* genes, and mixed at 1∶3 (0.25, lane 2), 1∶7 (0.12, lane 3) or 1∶15 (0.06, lane 4) ratios. Partially purified virions were immunoblotted for Vpx (Vpx) and p27 Capsid (CA p27). (B) Human monocyte-derived adherent macrophages (MF, panels 1–8), primary CD4^+^ T lymphocytes activated with PHA and IL-2 (CD4^+^, panels 9–16), and Jurkat T cells (JK, panels 17–24) were infected with normalized amounts of VSV-G pseudotyped single cycle SIVmac 239_(GFP)_ virions containing various amounts of wild type (panels 2–6, 10–14, 18–22), or Q76A (panels 7, 15, 23) and F80A (panels 8, 16, 24) substituted Vpx proteins, or mock infected (panels 1, 9 and 17). Cells were harvested 4 days (MF), or 2 days (CD4+ and JK), following infection and GFP expression in the transduced populations was analyzed by flow cytometry. Bivariant plots of GFP expression (abscissa) versus forward scatter (ordinate) are shown. Percent fractions of GFP-positive cells (boxed) are indicated. Of note, SIVmac 239_(GFP)_ containing wild type Vpx transduced between 5% and 60% of macrophages and Vpx enhanced transduction between 20-fold and 100-fold in 6 independent experiments, which probably reflects the donor-dependent variability of macrophage populations used.

VSV-G pseudotyped single cycle SIVmac 239_(GFP)_ viruses encoding wild type or mutated Vpx variants that do not bind VprBP, or possessing an inactive vpx coding sequence due to termination codon substitutions for methione codons M1 and M62 were produced from HEK 293T cells. All viruses contained a frameshift mutation in the *env* gene which prevented expression of a functional Env glycoprotein, and expressed GFP marker protein from an IRES element positioned immediately downstream of the *nef* gene (SIVmac 239_(GFP)_). A reference panel of virions containing decreasing amounts of wild type Vpx were also produced from HEK 293T cells transiently co-expressing SIVmac 239_(GFP)_ proviral construct possessing wild type *vpx* gene mixed with an isogenic construct containing the M1- and M62- mutated *vpx*, at 1∶3, 1∶7, or 1∶15 ratio. Virions were partially purified and concentrated by pelleting through 20% sucrose cushion and then analyzed by immunobloting for p27 Capsid and for Vpx. As shown in Figure 3A, the Q76A and F80A substitutions had only minor effects on the abilities of the mutant proteins to be incorporated into the virions (compare lanes 5 and 6 with 1–4). The H82R substituted Vpx was incorporated into viral particles very poorly (data not shown), and therefore was not studied further.

Next we measured the abilities of the VSV-G pseudotyped single cycle virions to transduce human monocyte derived adherent macrophages. Monocytes obtained from human peripheral blood mononuclear cells (PBMC) by negative selection for CD3, CD7, CD16, CD19, CD56, CD123 and Glycophorin were differentiated into macrophages in the presence M-CSF. Macrophage cultures were then infected with normalized virion preparations and transduction efficiencies of the wild type and mutant viruses were quantified by flow cytometric analysis of GFP expression in the infected cell populations. As controls, CD4^+^ T lymphocytes purified from PBMC by positive selection for CD4 and activated by phytohemagglutinin in the presence of IL-2, and Jurkat T cells, were also infected and analyzed in parallel. As shown in Figure 3B, panels 2–6, wild type Vpx stimulated macrophage transduction by up to 100-fold, in a dose-dependent manner (5.1% vs 0.06% GFP-positive cells). Significantly, Vpx_(Q76A)_, or Vpx_(F80A)_, substituted Vpx failed to support macrophage infection (panels 7 and 8), even though the mutant Vpx molecules were efficiently incorporated into the virions. In contrast, all viruses displayed similar infectivities to primary CD4^+^ T lymphocytes and Jurkat T cells (panels 9–16 and 17–24), indicating that Vpx is not required for transduction of primary T cells and established T cell lines, consistent with previous observations [2]. Thus, the Q76A and F80A changes link SIVmac Vpx ability to enhance macrophage transduction to its interaction with VprBP and its associated E3 ubiquitin ligase complex.

### HIV-2 Vpx also uses VprBP/DCAF1 to enable macrophage infection

**Figure 4:**
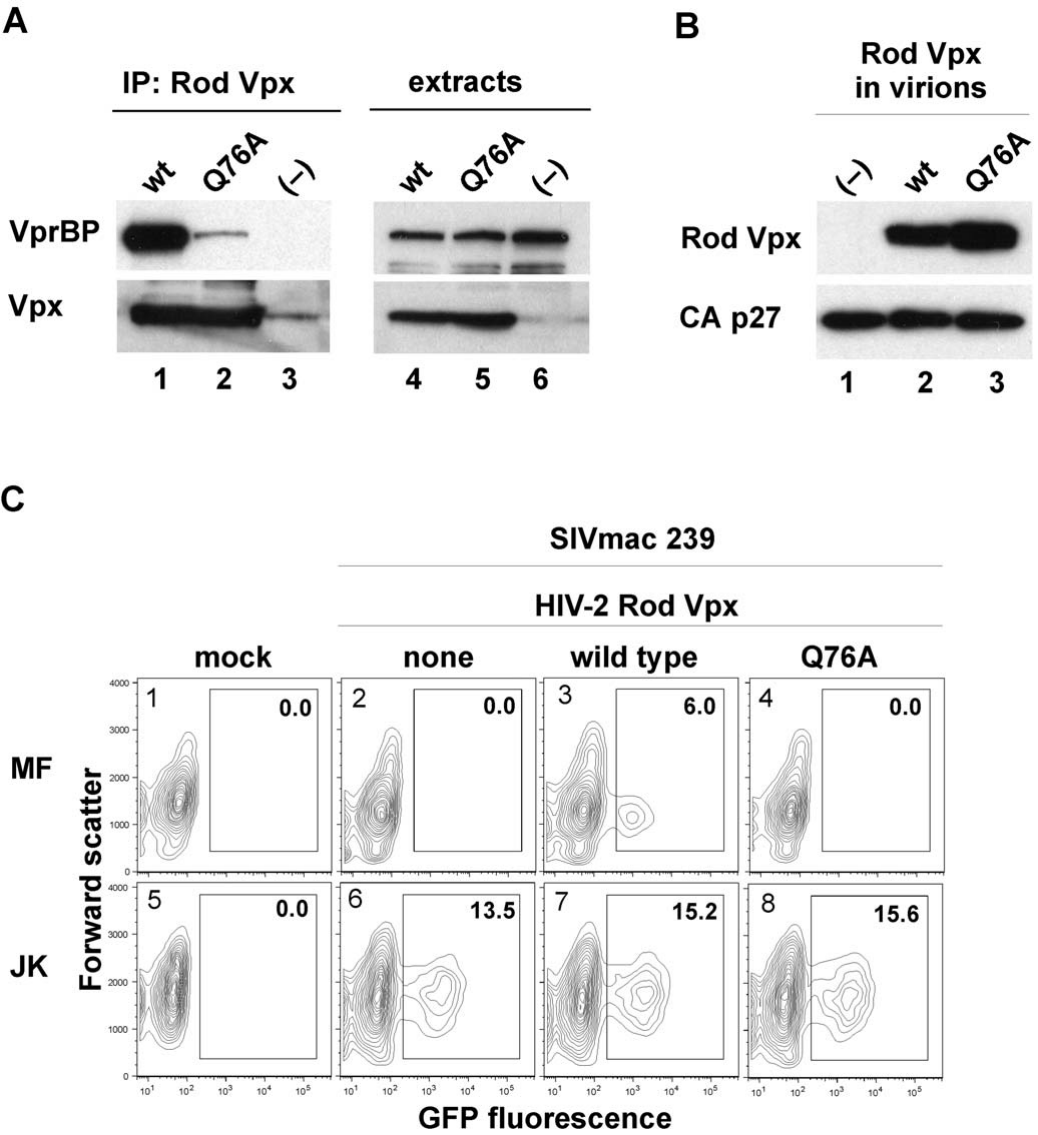
HIV-2 Rod Vpx facilitates macrophage transduction through its interaction with VprBP. (A) HIV-2 Rod Vpx binds VprBP/DCAF1. hfa-tagged wild type (lanes 1 and 4) or Q76A substituted (lane 2 and 5) HIV-2 Rod Vpx proteins were transiently expressed in HEK 293T cells and precipitated from detergent extracts with FLAG-M2 affinity resin. VprBP and hfa-Vpx were detected in immune complexes (left panel) and cell extracts (right panel) by immunoblotting and visualized by enhanced chemiluminescence. (B) Wild type and Q76A substituted HIV-2 Rod Vpx proteins are incorporated into SIVmac virions. VSV-G pseudotyped single cycle SIVmac 239_(GFP)_ virions were produced from HEK 293T cells transiently coexpressing wild type (lane 2) or Q76A mutated (lane 3) Rod Vpx, SIVmac 239_(GFP)_ proviral clone with disrupted *vpx* and *env* genes, and VSV-G. Virions were partially purified and analyzed for Vpx and p27 Capsid by Western blotting. (C) Human monocyte-derived macrophages (MF, panels 1–4), and Jurkat T cells (JK, panels 5–8) were infected with normalized amounts of viruses characterized in (B) above, or mock infected (panels 1 and 5), and GFP positive cells were determined after 4 days (MF), or 2 days (JK), by flow cytometry. Percent fractions of GFP-positive cells (boxed) are indicated.

Vpx proteins encoded by HIV-2 viruses also enhance transduction of monocyte-derived cells, but a previous report suggested that they may be unable to bind VprBP [22],[24]. This in turn raised a question whether HIV-2 Vpx uses a VprBP-independent mechanism to enable macrophage infection. To address this issue we asked whether Vpx variant encoded by HIV-2 Rod proviral clone binds VprBP. We chose this particular Vpx variant because it is required for the ability of HIV-2 Rod to transduce primary macrophages and, therefore, is functional [24]. Of note, it is evident from phylogenetic analyses that both the Rod Vpx and SIVmac 239 Vpx are representative of two major groups of HIV-2 Vpx variants (see Figure S2). As shown in Figure 4A, wild type, but not Q76A-substituted, Rod Vpx protein readily bound VprBP in a transient expression assay in HEK 293T cells (compare lanes 1 and 2). Next we assessed the abilities of both proteins to enhance macrophage transduction by a single cycle SIVmac 239_(GFP)_ reporter virus. We found that only wild type Rod Vpx rescued the infectivity of single cycle SIVmac 293_(GFP)_ reporter virions that were devoid of SIVmac Vpx, even though both the wild type and Q76A substituted Rod Vpx variants were incorporated into the virions to similar extents (Figure 4B and 4C). We conclude that the interaction with VprBP is a conserved function of SIVmac and HIV-2 Vpx proteins, and that both use VprBP to enable macrophage infection.

### Vpx uses VprBP to support reverse transcription of SIVmac in macrophages

**Figure 5:**
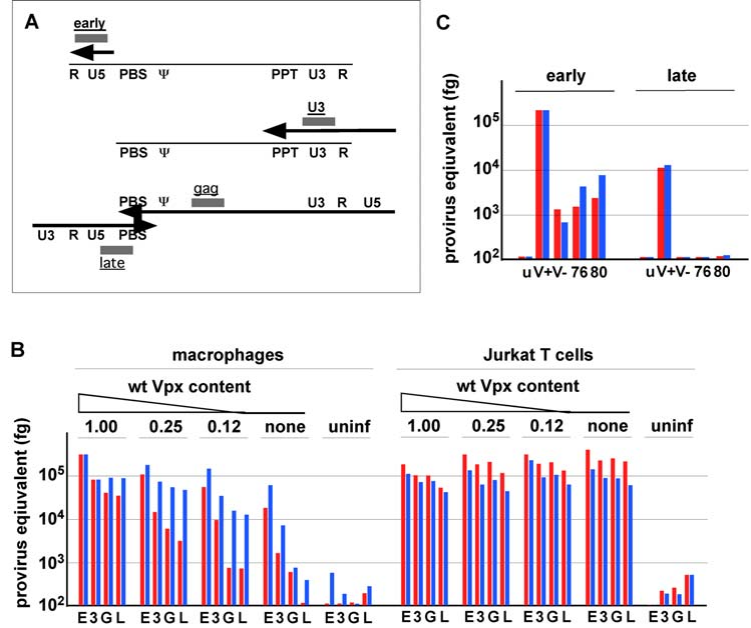
Reverse transcription of SIVmac 239_(GFP)_ virions comprising Vpx_(Q76A)_ and Vpx_(F80A)_ in macrophages is compromised. (A) Location of reverse transcription intermediates used to gauge the progression of reverse transcription of SIVmac 239_(GFP)_ genome. Regions amplified by “early”, “U3, “gag” and “late” sets of oligonucleotide primers are represented by grey boxes. The thin line represents viral RNA and the locations of the R, U5, primer binding site (PBS), packaging signal (Ψ), polypurine tract (PPT) and U3 regions are indicated. The thick arrows represent viral cDNA. (B) Steady state levels of reverse transcription intermediates following infection with VSV-G pseudotyped single cycle SIVmac 239_(GFP)_ virions deficient in Vpx. Macrophages and Jurkat T cells were infected with the reference set of SIVmac 239_(GFP)_ virions containing various amounts of wild type Vpx, characterized in Figure 3. DNA was prepared from the infected cells 18 hour or 72 hours following infection and 50 ng aliquots were analyzed in duplicate by real time PCR with oligonucleotide primers that recognize “early”(E), “U3”(3), “gag”(G) and “late”(L) reverse transcription products, as indicated below the histograms. The amounts of reverse transcription products were calculated by comparison to standard curves generated with serially diluted SIVmac 239 proviral DNA and are shown in red and blue for the 18-hour and 72-hour timepoint, respectively. The variances between duplicate data points were less than 4%. (C) Q76A and F80A substitutions disrupt Vpx function in macrophages. Macrophages were not infected (u) or infected with single cycle SIVmac 239_(GFP)_ virions containing Q76A (76) or F80A (80) substituted, wild type (V+), or no Vpx (V−), characterized in Figure 3A, and reverse transcription was analyzed with “early” and “late” primers as described above.

Vpx was reported to be essential for efficient reverse transcription and/or nuclear import of lentiviral genomes in monocyte-derived cells [3],[8]. Hence we examined the effect of Q76A and F80A substitutions in Vpx on reverse transcription (RT) of the incoming SIVmac genomes by real-time quantitative fluorescent PCR. Macrophages were transduced with a reference panel of VSV-G pseudotyped single cycle SIVmac 239_(GFP)_ virions containing decreasing amounts of wild type Vpx, or Vpx_(Q76A)_ and Vpx_(F80A)_ variants, characterized in Figure 3A. DNA was isolated from the transduced cells 18 hours and 72 hours later and RT intermediates were quantified by real time PCR with four sets of primers shown in Figure 5A. The primers were designed to amplify strong-stop DNA (early), RT products synthesized immediately following minus strand transfer (U3), or a region of the *gag* gene located approximately 8000 nucleotides distal from U3 (gag), as well as late RT products synthesized following successful plus strand transfer (late). As shown in Figure 5B, these analyses revealed that reverse transcription was defective following infection with virions lacking, or containing suboptimal amounts of Vpx. First, the steady state levels of the early strong-stop RTs were approximately 10-fold lower in the absence of Vpx and the magnitude of the decrease was inversely correlated with the Vpx virion content. Second, the levels of U3, gag and late RTs were progressively lower upon infection with Vpx-deficient virions (approx 100-fold, 300-fold and 1000-fold, respectively) at the 18 hour time point. These differences were less pronounced at the 72 hour time point. Importantly, Vpx was not required for efficient reverse transcription following infection of Jurkat T cells. Together, these observations indicate that Vpx is required for events that lead to an efficient initiation and progression of reverse transcription of SIVmac genome in macrophages.

Next we tested the effects of Q76A and F80A substitutions in SIVmac Vpx for its ability to enable efficient reverse transcription of the SIVmac genome in macrophages. As shown in Figure 5C, both Vpx variants conferred a Vpx-deficient virion phenotype. Since Q76A and F80A each disrupts Vpx binding to VprBP, these findings link the interaction with VprBP to Vpx ability to facilitate reverse transcription of lentiviral genome in macrophages.

### VprBP mediates macrophage transduction by SIVmac

**Figure 6:**
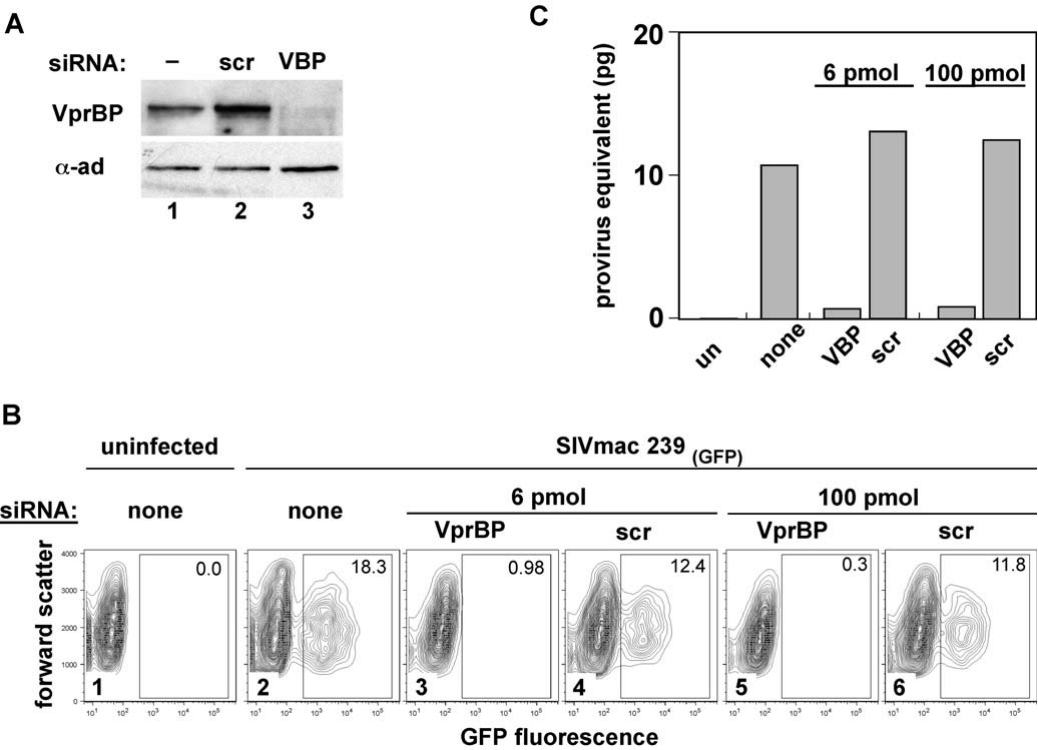
VprBP is important for the ability of SIVmac 239 to transduce macrophages. (A) Depletion of VprBP levels in macrophages by RNAi. Detergent extracts prepared from macrophages (lane 1) transfected with a control non-targeting siRNA (lane 2, scr) or siRNA pool targeting human VprBP (lane 3, VBP) four days after initiation of RNAi were analyzed by immunoblotting with rabbit anti-VprBP IgG, or with an antibody to the α-subunit of the AP-2 clathrin adaptor complex (α-ad), to control for equal loading. (B) and (C) VprBP-depleted macrophages resist SIVmac 239 infection. Macrophages transfected with the indicated amounts of VprBP-targeting (panels 3 and 5) or non-targeting (panels 4 and 6) siRNA pools and nontransfected macrophages (panel 2) were infected with VSV-G pseudotyped SIVmac 239_(GFP)_ virus two days after initiation of RNAi. Flow cytometric analysis of GFP expression (B) and real time PCR quantification of gag DNA (C) in the transduced populations were performed 3 days later.

To obtain further insight into the role of VprBP, we knocked down its expression in macrophages by RNA interference (RNAi, [25]). As illustrated in Figure 6A, a pool of small interfering RNAs (siRNA) targeting VprBP, but not the control non-targeting siRNAs, severely diminished VprBP expression (compare lane 3 with 1 and 2). Two days following initiation of RNAi macrophages were infected with VSV-G pseudotyped single cycle SIVmac 239_(GFP)_ reporter virus and the transduction efficiency was assessed 3 days later. Flow cytometry analysis of GFP expression revealed that nontargeting siRNA decreased transduction efficiencies by only approximately 30% and the magnitude of this effect was constant over a wide range of siRNA concentrations (Figure 6B, compare panels 4 and 6 with 2). A similar result was observed with another non-targeting siRNA pool (data not shown). These observations indicate that non-specific engagement of RNAi machinery had only a minor negative effect on macrophage transduction by SIVmac 239. In contrast, RNAi to VprBP decreased transduction efficiency by approximately 10-fold at a lower dose, and 30-fold at a higher dose of the targeting siRNA (compare panel 3 with 4 and 5 with 6). These experiments were repeated 4 times and we consistently observed a decrease in transduction efficiency following RNAi to VprBP, ranging between 6-fold and >100-fold. To further exclude the possibility that the observed resistance of VprBP-depleted macrophages to SIVmac infection is caused by the off target effects of the siRNA pool targeting VprBP, additional experiments were performed using individual VprBP-specific siRNAs (Figure S3). We observed good correlation between the abilities of the four siRNAs to knock down VprBP expression and to disrupt macrophage transduction by SIVmac 239_(GFP)_ reporter virus. Of note, VprBP-depletion in U2OS cells did not compromise transduction of these cells by SIVmac 239_(GFP)_ reporter virus regardless of the presence or absence of Vpx (Figure S4). Together these data indicate that VprBP is required for efficient macrophage transduction by SIVmac.

If VprBP indeed facilitates macrophage transduction through the interaction with Vpx, we expected the arrest of SIVmac replication in the absence of VprBP and that in the absence of Vpx to be similar in nature. To test this prediction we examined the steady state levels of SIVmac 239_(GFP)_ late reverse transcription products 72 hours post infection of VprBP-depleted and control macrophage populations, by real time PCR. As expected, the levels of late reverse transcripts were approximately 100-fold lower in VprBP-depleted versus non-targeting siRNA treated, or untreated macrophages (Figure 6C, compare VprBP to scr, or none). Together our data indicate that VprBP has an important role in macrophage transduction by SIVmac and that this function requires Vpx.

## Discussion

Vpx enables efficient transduction of monocyte-derived cells, such as macrophages and DC's by SIVsm/mac and HIV-2 viruses; however the mechanism that mediates this effect has not been identified. Our findings link this Vpx function to its ability to interact with components of the ubiquitin proteasome system and identify a ternary protein complex - comprising DDA1, DDB1 and VprBP/DCAF1, a putative substrate receptor for Cullin 4-based E3 ubiquitin ligase - as the immediate downstream effector that Vpx uses to promote macrophage transduction. Importantly, the DDB1-VprBP/DCAF1 module was previously shown to participate in a functional Cullin 4 E3 ubiquitin ligase complex [13]. Together, these findings support a model in which Vpx usurps the Cullin 4 E3 ubiquitin ligase utilizing the VprBP/DCAF1 to overcome a block to lentivirus replication upon its entry into monocyte-derived cells.

Our data indicate that Vpx acts early following virion entry into macrophages to allow efficient initiation as well as completion of reverse transcription of the incoming SIVmac RNA genomes. This can be clearly seen from a >10-fold decrease in steady state levels of early reverse transcription products and 10^3^-fold decrease in late reverse transcripts upon challenge with Vpx-deficient virions. These phenotypes could result from defects in virion uncoating and/or in its transit into a permissive cytoplasmic compartment.

A similar, albeit less dramatic, loss of *vpx* function phenotypes were previously reported for other SIVsm/mac viral isolates and/or *vpx* alleles upon infection of monocyte-derived DCs [8]. The finding that Vpx is required for efficient reverse transcription in macrophages was somewhat surprising, because it has been thought that this factor acts at a later stage in the replication cycle by enabling the import of the fully reverse transcribed preintegration complex into the nucleus [3]. Our findings, taken together with these previous observations, indicate that SIVmac replication is restricted by the same mechanism in DCs and in macrophages. Thus, it is important to refocus future studies towards post entry events that precede reverse transcription in these monocyte derived cells.

The phenotype of Vpx-deficient virions is reminiscent of that resulting from a block to retrovirus replication imposed by tripartite motif protein 5α (TRIM5α) restriction factors. TRIM5α is a E3 ubiquitin ligase that inactivates the incoming virions, probably by deregulating their uncoating so rapidly that the late reverse transcripts fail to accumulate [26],[27],[28]. Also, the observation that proteasome inhibitors partially rescue reverse transcription of Vpx-deficient viruses in DCs is consistent with the idea that SIVmac virions may be targeted by a TRIM5α-like restriction, or by another E3 ubiquitin ligase in monocyte-derived cells [8]. Whereas these observations raise the possibility that Vpx could act by counteracting TRIM5α, we note that this is not likely, because TRIM5α is expressed in Jurkat T cells [29], which we found not to restrict Vpx-deficient SIVmac virions.

How does Vpx facilitate reverse transcription in macrophages via its interaction with VprBP? As mentioned above, a recent study suggested that the replication of SIVmac cells could be restricted, at least in part, by an as yet unidentified E3 ubiquitin ligase [8]. We initially considered that VprBP-linked Cullin 4 E3 complex could be that enzyme and that Vpx counters the restriction by inhibiting its activity. However, our data from RNAi experiments revealed that VprBP is not required for the restriction to occur and, therefore, do not support this possibility. Furthermore, the incoming virions probably contain at most only several hundred Vpx molecules, similar to Vpr, which also is virion recruited through its interaction with Gag p6 [6],[30],[31]. Therefore, it is difficult to envision that the limited amounts of virion-bound Vpx would be able to saturate and inhibit the cellular pool of VprBP-associated Cullin 4 E3 complexes, even by a noncompetitive mechanism.

Instead of blocking SIVmac replication, our evidence indicates that VprBP is required for Vpx to overcome the block, implying that Vpx uses VprBP-associated E3 to enable reverse transcription in macrophages. Notably, the same VprBP-associated ubiquitin ligase was shown previously to be targeted by a Vpx paralogue, Vpr, which stimulates the intrinsic catalytic activity ofthis E3 [13]. The findings that both Vpx and Vpr interact with VprBP in a similar manner via their C-terminal regions, and that both interactions lead to post-translational modification of their associated Cullin 4 subunits suggest that Vpx also usurps the VprBP-associated E3, probably to inactivate a cellular factor that inhibits lentivirus replication in macrophages and DC's. Indeed, viral accessory proteins are known to utilize E3 ubiquitin ligases to direct ubiquitination and proteasomal degradation of cellular proteins that mediate innate immunity to viral infection [32].

Both Vpx and Vpr bind VprBP through similar molecular interactions, yet the functional outcomes are different. Vpr uses VprBP-associated E3 to activate DNA damage checkpoint controlled by the Ataxia-telangiectasia and Rad3-related (ATR) kinase, while Vpx does not have this function and, instead, enables efficient reverse transcription of SIVmac genome in monocyte-derived cells [33]. These different outcomes likely reflect that Vpr and Vpx recruit different sets of substrates for ubiquitination by the same E3 enzyme [34],[35], and that they affect differently the activities of their associated Cullin 4 E3s (see Figure S1). It will be important in the future to identify cellular proteins whose ubiquitination is altered by Vpx and Vpr in order to advance the understanding of these important virulence factors.

In summary, our findings provide novel insights into the mechanism by which Vpx enables macrophage infection, as they link this function to Vpx interaction with VprBP and its associated Cullin 4 E3 ubiquitin ligase complex. Further studies of how Vpx manipulates protein ubiquitination through its interaction with VprBP should lead to detailed understanding of the biochemical mechanism that limit replication of primate lentiviruses in monocyte-derived cells, and how it is countered by viruses of the HIV-2/SIVmac/sm lineages. This knowledge in turn will likely lead to the conception of new strategies aimed to prevent the virus from establishing reservoirs in these cells.

## Materials and Methods

### Expression plasmids

pCG expression vectors expressing epitope tagged VprBP/DCAF1, DDB1, DDA1, Cullin 4A, and Vpr proteins of HIV-1 NL43 and SIVmac 239 viruses were described previously [13]. SIVmac 239 Vpx was tagged with hfa- triple epitope tag and subcloned into BABE(puro) and pCG vectors [13]. HIV-2 Rod *vpx* gene was amplified by PCR from Rod proviral clone [24] kindly provided by Michael Emerman (Fred Hutchinson Cancer Research Center, Seatte). Mutations were introduced using QuikChange XL II kit (Stratagene, La Jolla, CA, United States) and confirmed by DNA sequencing.

### Transient transfections of HEK 293T cells, immunoprecipitations, and immunoblotting

HEK 293T cells were transfected by calcium phosphate co-precipitation method. Detergent extracts and anti-FLAG immune complexes were analyzed by immunobltting as described previously [36]. FLAG-, HA- and myc- epitope tagged proteins were detected with anti-FLAG M2 (Sigma-Aldrich, St. Louis, MO, United States), 12CA5, and 9E10 monoclonal antibodies (mAb), respectively. The following antibodies were also used: anti-DDB1 (37-6200) from Zymed (Invitrogen, Carlsbad, CA, United States), anti-α-adaptin (AC1-M11) from Alexis Corp (San Diego, CA, United States), anti-Gag SIVmac 251 (13-112-100) from Advanced Biotechnologies Inc. (Columbia, MD, United States) and anti-Vpx 6D2.6 Vpx hybridoma supernatant. DDA1 and VprBP were detected with rabbit sera raised to recombinant proteins [13].

### Immunoaffinity purification of epitope-tagged proteins and MudPIT analysis

SIVmac 239 Vpx and its associated proteins were purified from U937 cells stably expressing hfa-tagged Vpx, or HEK 293T cells transiently expressing hfa-Vpx by two sequential immunoprecipitations via FLAG and HA epitope tags, each followed by competitive elution with the appropriate peptide epitope. MudPIT analysis was performed as described previously [13]. Tandem mass (MS/MS) spectra were interpreted using SEQUEST [37] against a database of 82242 sequences, consisting of hfa-tagged SIVmac Vpx, usual contaminants, and 40873 human proteins, as well as, to estimate false discovery rates, randomized amino acid sequences derived from each non-redundant protein entry. Peptide hits from multiple runs were compared using CONTRAST [38].

### SIVmac 239 proviral clones and viruses

Vpx and Vpr mutations were introduced into a single cycle SIVmac 239_(GFP)_ reporter proviral clone containing a frameshift mutation in the *env* gene constructed by filling in a unique ClaI site [39]. A proviral clone deficient for *vpx* was constructed by substituting methionine and serine codons at positions 1 and 2 in *vpx* with threonine and termination codons, respectively, such as not to alter the overlapping *vif* gene. The second consecutive methionine codon (M62) in *vpx* was also changed to a termination codon to prevent the possibility that a truncated C-terminal fragment of Vpx protein will be expressed. Mutagenesis was performed with QuikChange XLII kit (Stratagene) using 1.3 kb PacI-SphI fragment of SIVmac 239_(GFP)_ provirus [39] that comprises *vif* and *vpx* open reading frames, subcloned into pCR 2.1 vector, as a template. All mutations were confirmed by DNA sequencing and reintroduced into SIVmac 239_(GFP)_ proviral clones containing a frameshift mutation in *env*, by exchanging the 1.3 kb PacI-Sph1 restriction fragment. VSV-G pseudotyped single cycle viruses were produced from HEK 293T cells transiently transfected with proviral clones and a VSV-G expression plasmid. In some experiments *vpx*-defective SIV were complemented in trans with wild-type or mutant Vpx proteins expressed from cotransfected pCG vectors. Culture medium was harvested 24 hours after transfection, cell debris removed by centrifugation at 7,000 rpm for 10 minutes and virus containing supernatants were then treated with DNAse I (Roche) for 60 minutes at 30°C. Viral particles were partially purified and concentrated by pelleting through 20% sucrose in 10 mM Tris-HCl [pH 7.4], 100 mM NaCl, 1 mM EDTA cushion at 27,000 rpm for 3 hours. Virion preparations were normalized based on reverse transcriptase assays and/or infectivity to Jurkat T cells, and stored at −70°C.

### Preparation of macrophage and CD4^+^ T cells, infections and flow cytometry analysis

Monocytes obtained from human PBMCs by negative selection for CD3, CD7, CD16, CD19, CD56, CD123 and Glycophorin using Monocyte Isolation Kit II (Miltenyi Biotec Inc., Auburn, CA, United States), were plated in 24 well plates at 4–7×10^5^ cells/well and differentiated into macrophages by culturing in DMEM supplemented with 10% fetal bovine serum (FBS), Macrophage-Colony Stimulating Factor (M-CSF, 50 ng/ml, R&D Systems, Minneapolis, MN, United States) for 6 days. Cells were fed every alternate day by replacing one half of the cell culture medium with fresh medium. Purity of CD14^+^ cells obtained by negative selection with the Monocyte Isolation Kit II from Miltenyi usually ranged between 95% and 99% while the final purity of the adherent macrophage population was typically greater than 99.9%. RNA interference was initiated at day 6 and followed by infections with SIVmac on day 8. Cells were harvested for QPCR analysis of reverse transcription products 18 hours to 72 hours post infection. Flow cytometry analysis of GFP expression was performed 4 days post infection. Macrophages were detached from wells by trypsin treatment, resuspended in 1% paraformaldehyde and GFP expression analyzed by flow cytometry. CD4^+^ T cells were purified from PBMC using CD4^+^ T cell isolation kit (Miltenyi Biotec) and stocks were frozen in 10^7^ cell aliquots. Stocks were plated in 5 ml of RPMI 1640 supplemented with 10% FBS, 2 mM glutamine, 10 mM HEPES, pH = 7.4, 50 µM β-mercaptoethanol, and containing phytohemagglutinin (PHA, 10 µg/ml) and recombinant human IL-2 (10 u/ml, Roche) in single wells of a 6 well plate. After 48 hours cultures were diluted into the same medium but without PHA and 5×10^5^ cell aliquots were infected in the total volume of 2 ml in wells of a 24 well plate. Expression of GFP marker protein was quantified 48 hours post infection by flow cytometry.

### Real time PCR

SIV reverse transcription products were quantified by real time PCR on ABI PRISM 7700 SDS. A typical reaction contained 50 ng of DNA isolated with DNAeasy Kit (Qiagen, Valencia, CA, United States) from infected or control cells and SYBR Green PCR master mix in a total volume of 25 µl (Applied Biosystems, Foster City, CA, United States). Early reverse transcription products were amplified with ERT.2.s (5′-CTTGCTTGCTTAAAGCCCTCTT-3′) and S.ERT.as (5′-CAGGGTCTTCTTATTATTGAGTACC-3′) primers, U3 with U3.SIV.s (5′-ATCATACCAGATTGGCAGGATT-3′) and U3.SIV.as (5′-GAAGTTTGAGCTGGATGCATTA-3′), gag with SIV.GAG.s (5′-ATTAGTGCCAACAGGCTCAGA-3′) and SIV.GAG.as (5′-GCATAGTTTCTGTTGTTCCTGTTT-3′), late with SIV.1 (5′-AGCTAGTGTGTGTTCCCATCTC-3′) and SIV.3 (5′-TACTCAGGAGTCTCTCACTCTCCT-3′).

Serial dilutions of known amounts of a plasmid containing SIVmac293 provirus, served as a copy number standard to generate standard curves.

### RNA interference

Macrophages cultured in 24 well plates (Becton & Dickinson, San Jose, CA, United States) were fed with antibiotic free 10% serum containing DMEM 24 hrs before transfections. Cells were transfected with 1–200 pmol aliquots of a control nontargeting pool of siRNA (D-001206-14-05, Dharmacon, Lafayette, CO, United States) or ON-TARGET plus SMARTpool siRNA targeting human VprBP (L-021119-01), or individual VprBP-specific siRNAs (VprBP1 sense: GAUGGCGGAUGCUUUGAUAUU, antisense: UAUCAAAGCAUCCGCCAUCUU; VprBP2 sense: GGAGGGAAUUGUCGAGAAUUU, antisense: AUUCUCGACAAUUCCCUCCUU; VprBP3 sense: ACACAGAGUAUCUUAGAGAUU, antisense: UCUCUAAGAUACUCUGUGAUU; VprBP10 sense: CCACAGAAUUUGUUGCGCAUU, antisense: UGCGCAACAAAUUCUGUGGUU) using Lipofectamine 2000 (Invitrogen) according to manufacturer's instructions. Briefly, siRNA stocks were prepared in phosphate buffered saline (PBS). Liposomes were formed using 4 µl of Lipofectamine 2000 per well, as recommended by manufacturer (Invitrogen). 4–6 hours post transfection culture medium was replaced with fresh antibiotic-free DMEM supplemented with 10% FBS. U2OS cells were plated at 5×10^4^/well of 12 well plate in 1 ml of DMEM supplemented with 10% FBS and 2 mM glutamine in the absence of antibiotics 24 hours before initiation of RNAi. Cells were transfected with lipofectamine 2000 (4 µl/well) containing indicated amounts of siRNA duplexes in 0.5 ml of medium, and the medium was replaced with 1 ml of fresh medium 5 hours later. 48 hours following initiation of RNAi cells were harvested for immunoblot analysis of VprBP expression, or infected with SIVmac or HIV-1 derived vectors. Transduction efficiencies were quantified by flow cytometric analysis of GFP expresssion.

## Supporting Information

Figure S1 Characterization of Vpx-associated Cullin 4 E3 complex. (A) SIVmac Vpx and HIV-1 NL43 Vpr induce post-translational modification of Cullin 4. Myc-tagged Cullin 4A (m-Cul4) was expressed alone (lane 1), or together with FLAG-tagged SIVmac 239 Vpx (f-Vpx, lane 2), or HIV-1 NL43 Vpr (f-Vpr(H), lane 3) in HEK 293T cells. Ectopically expressed Cullin 4A and Vpr/Vpx were detected in detergent extracts with anti-myc- or anti-FLAG- epitope antibodies, respectively. (B) In vitro intrinsic ubiquitin ligase activities of SIVmac Vpx and HIV-1 NL43 Vpr -associated E3 complexes. Cul4-DDB1[VprBP] E3 complexes were assembled in the absence (lanes 1, 2) or in the presence (lanes 3, 4) of Vpx, or Vpr (lanels 5, 6), in HEK 293T cells and purified by immunoprecipitation via their FLAG-tagged VprBP subunits (13). Protein complexes were incubated with E1 and ubiquitin in the presence, or absence, of E2 as indicated. Cullin 4A and its ubiquitinated forms were detected by immunoblotting for Cullin 4.
(0.38 MB TIF)

Figure S2 Phylogenetic relationship between SIVmac 239 and HIV-2 Vpx protein variants. (A) Unrooted phylogenetic tree was constructed using Vpx amino acid sequences encoded by fully sequenced HIV-2 viruses found in Genbank database in December 2007 (identified by their GenBank accession numbers), and CLUSTALW and Phylip software. SIVmac 239 Vpx was also included in the analysis. To more accurately reflect Vpx diversity, closely related sequences such as those of multiple virus isolates from the same individual were considered redundant and excluded from the analysis. SIVmac 239 and HIV-2 Rod Vpx variants are highlighed. (B) Multiple sequence alignment of HIV-2 Vpx amino acid sequences. Consensus HIV-2 Vpx amino acid sequence is shown in the top line. SIVmac 239 Vpx amino acid sequence is also included for comparison. Amino acid residues corresponding to those found to be critical for the interaction of SIVmac 239 Vpx with DDA1-DDB2-VprBP complex (Q76, F80, H82) are boxed. (.) indicate amino acid identity and (-) represent gaps introduced for optimal sequence alignment.
(0.36 MB TIF)

Figure S3 siRNA mediated inhibition of macrophage transduction by SIVmac 239 correlates with depletion of VprBP expression levels. Experiments were performed to correlate the ability of siRNA duplexes to knock-down VprBP expresion and to disrupt macrophage transduction by SIVmac 239. (A) RNAi was performed in U2OS cells with four individual siRNAs to VprBP: VprBP1, VprBP2, VprBP3 and VprBP10 and with a nontargeting siRNA (scr) as a negative control, at 0.5 pmol/well in 12 well plates. VprBP expression levels were assessed by immunoblotting 2 days after initiation of RNAi. Cell extracts were also probed with antibody specific for α-adaptin subunit of the AP-2 clathrin adaptor complex to confirm equal loading. HEK 293T cells transiently overexpressing VprBP were used as a positive control (lane 8). (B). Macrophages were infected with VSV-G pseudotyped single round SIVmac 239_(GFP)_ reporter viruses two days following the initiation of RNAi, and GFP marker expression was analyzed 4 days later by flow cytometry.
(0.60 MB TIF)

Figure S4 VprBP/DCAF1 is not important for the ability of SIVmac 239 to transduce U2OS cells. RNAi to VprBP was performed in U2OS cells with 0.5, 2.5 and 10 pmol of VprBP10 siRNA, or 2.5 and 10 pmol of nontargeting control siRNA/well in 12 well plates. 2 days after initiation of RNAi cells were (A) harvested for immunoblot analysis of VprBP expression levels, or (B) infected with VSV-G pseudotyped SIVmac 239_(GFP)_ reporter viruses possessing wild type Vpx (Vpx+), or not (Vpx−). GFP-positive cells was quantified by flow cytometry two days later. Transduction efficiencies were normalized to those seen with control U2OS cells that were not subjected to RNAi. Labels at the bottom of the histogram indicate data from U2OS cell populations that have not been subjected to RNAi (n), were treated with 10 pmol of nontargeting siRNA (c), or with 0.5 (1), 2.5 (2) and 10 (3) pmol of VprBP10 siRNA/well in 12 well plates.
(0.45 MB TIF)
