# Supplementary figures and images for: Lentiviral Vpx Accessory Factor Targets VprBP/DCAF1 Substrate Adaptor for Cullin 4 E3 Ubiquitin Ligase to Enable Macrophage Infection
Source: PLoS Pathog. 2008 May 9;4(5):e1000059. doi: 10.1371/journal.ppat.1000059 (PMC2330158; doi:10.1371/journal.ppat.1000059)

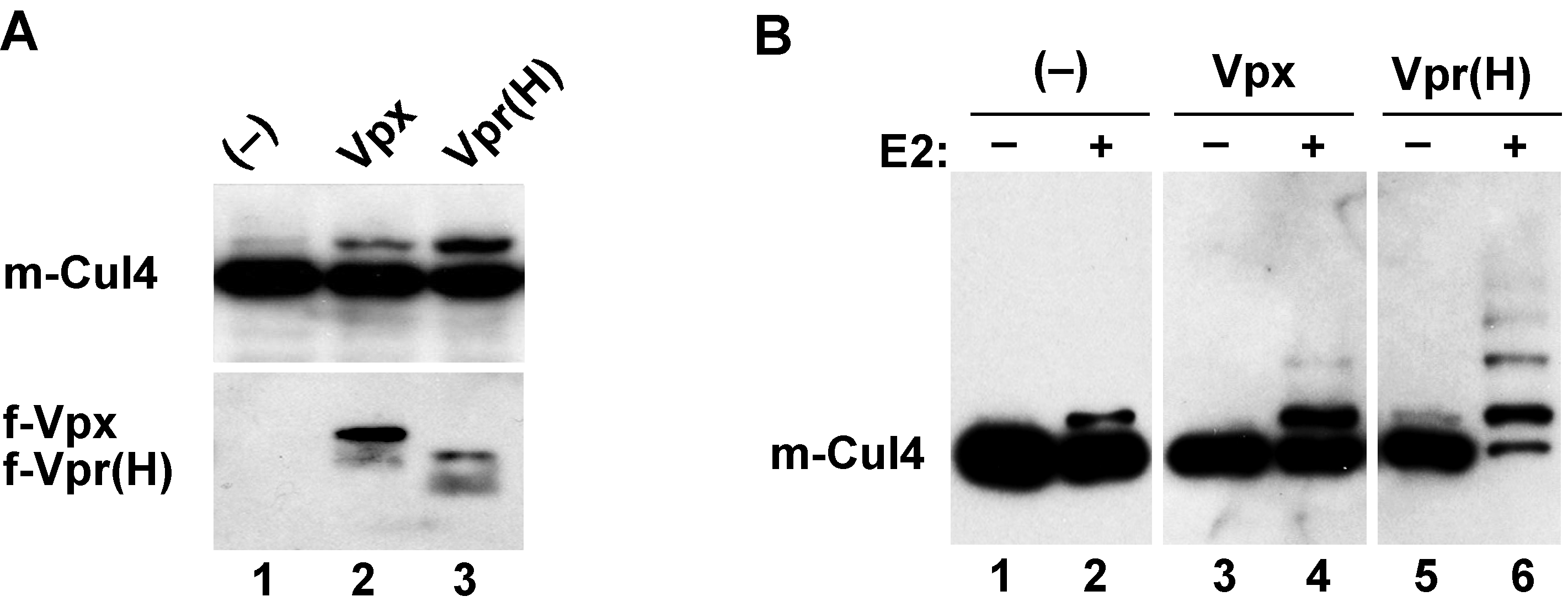

Supplement: Figure S1 — Characterization of Vpx-associated Cullin 4 E3 complex. (A) SIVmac Vpx and HIV-1 NL43 Vpr induce post-translational modification of Cullin 4. Myc-tagged Cullin 4A (m-Cul4) was expressed alone (lane 1), or together with FLAG-tagged SIVmac 239 Vpx (f-Vpx, lane 2), or HIV-1 NL43 Vpr (f-Vpr(H), lane 3) in HEK 293T cells. Ectopically expressed Cullin 4A and Vpr/Vpx were detected in detergent extracts with anti-myc- or anti-FLAG- epitope antibodies, respectively. (B) In vitro intrinsic ubiquitin ligase activities of SIVmac Vpx and HIV-1 NL43 Vpr -associated E3 complexes. Cul4-DDB1[VprBP] E3 complexes were assembled in the absence (lanes 1, 2) or in the presence (lanes 3, 4) of Vpx, or Vpr (lanels 5, 6), in HEK 293T cells and purified by immunoprecipitation via their FLAG-tagged VprBP subunits (13). Protein complexes were incubated with E1 and ubiquitin in the presence, or absence, of E2 as indicated. Cullin 4A and its ubiquitinated forms were detected by immunoblotting for Cullin 4. (0.38 MB TIF) [file ppat.1000059.s001.tif]

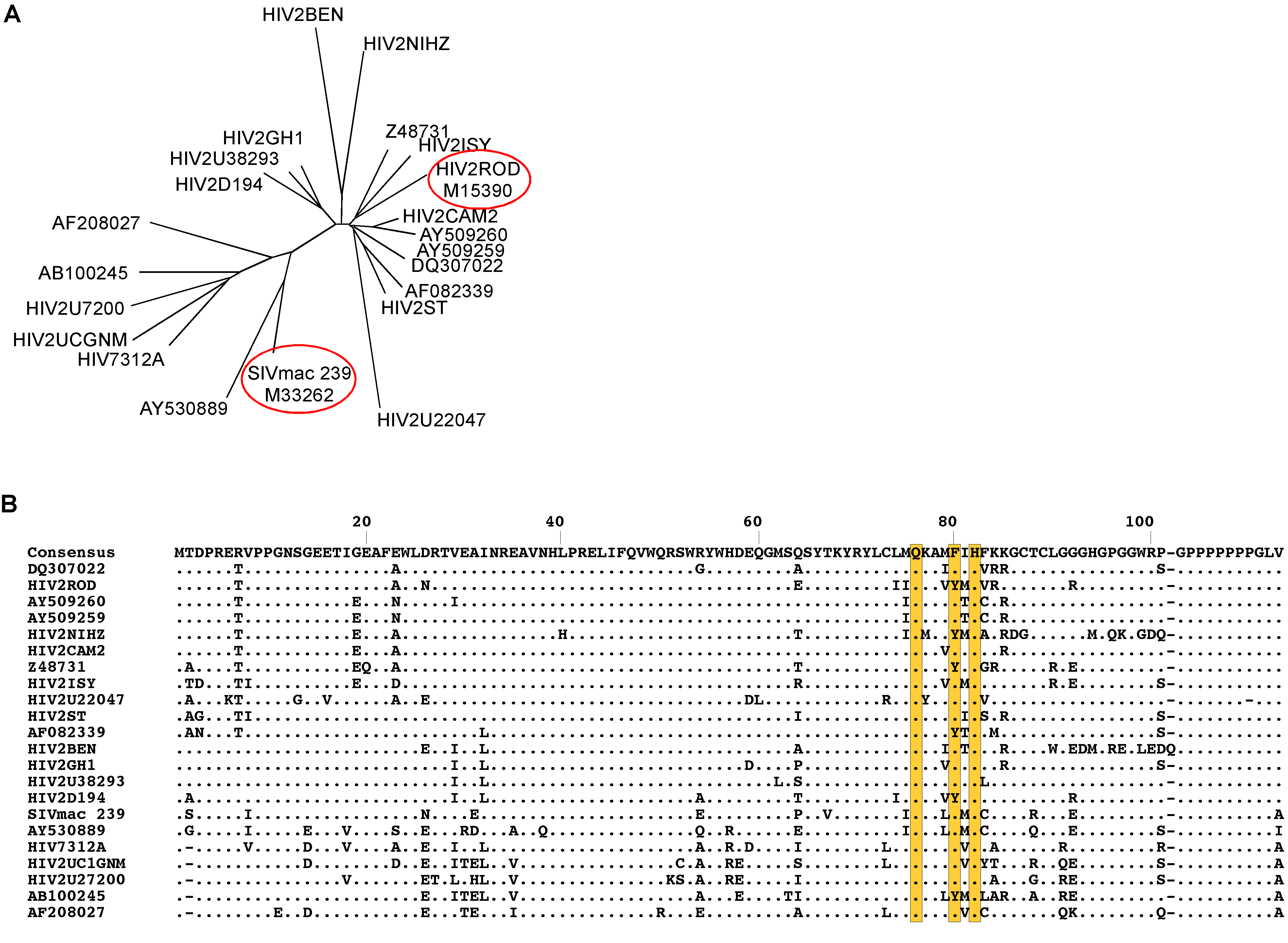

Supplement: Figure S2 — Phylogenetic relationship between SIVmac 239 and HIV-2 Vpx protein variants. (A) Unrooted phylogenetic tree was constructed using Vpx amino acid sequences encoded by fully sequenced HIV-2 viruses found in Genbank database in December 2007 (identified by their GenBank accession numbers), and CLUSTALW and Phylip software. SIVmac 239 Vpx was also included in the analysis. To more accurately reflect Vpx diversity, closely related sequences such as those of multiple virus isolates from the same individual were considered redundant and excluded from the analysis. SIVmac 239 and HIV-2 Rod Vpx variants are highlighed. (B) Multiple sequence alignment of HIV-2 Vpx amino acid sequences. Consensus HIV-2 Vpx amino acid sequence is shown in the top line. SIVmac 239 Vpx amino acid sequence is also included for comparison. Amino acid residues corresponding to those found to be critical for the interaction of SIVmac 239 Vpx with DDA1-DDB2-VprBP complex (Q76, F80, H82) are boxed. (.) indicate amino acid identity and (-) represent gaps introduced for optimal sequence alignment. (0.36 MB TIF) [file ppat.1000059.s002.tif]

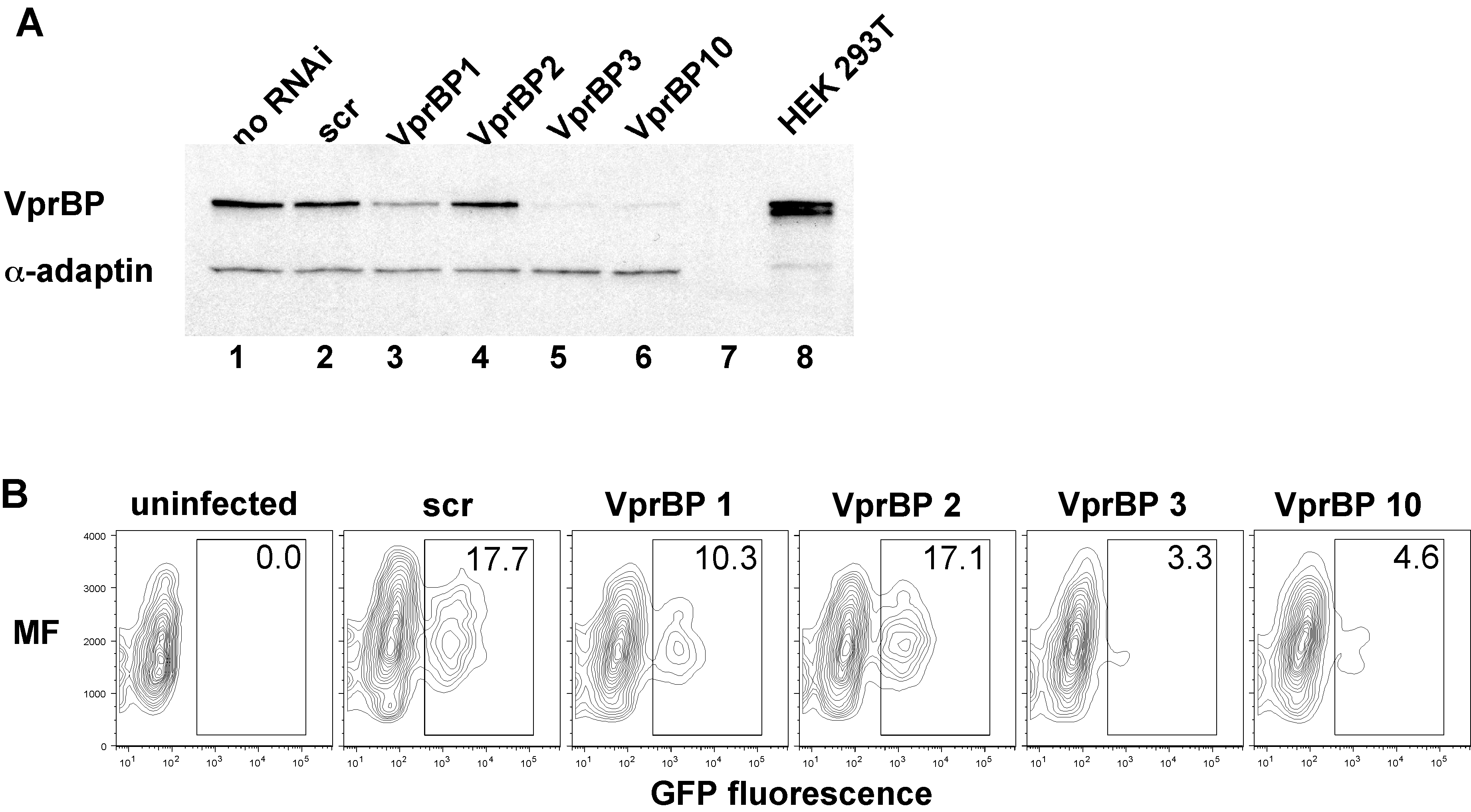

Supplement: Figure S3 — siRNA mediated inhibition of macrophage transduction by SIVmac 239 correlates with depletion of VprBP expression levels. Experiments were performed to correlate the ability of siRNA duplexes to knock-down VprBP expresion and to disrupt macrophage transduction by SIVmac 239. (A) RNAi was performed in U2OS cells with four individual siRNAs to VprBP: VprBP1, VprBP2, VprBP3 and VprBP10 and with a nontargeting siRNA (scr) as a negative control, at 0.5 pmol/well in 12 well plates. VprBP expression levels were assessed by immunoblotting 2 days after initiation of RNAi. Cell extracts were also probed with antibody specific for α-adaptin subunit of the AP-2 clathrin adaptor complex to confirm equal loading. HEK 293T cells transiently overexpressing VprBP were used as a positive control (lane 8). (B). Macrophages were infected with VSV-G pseudotyped single round SIVmac 239(GFP) reporter viruses two days following the initiation of RNAi, and GFP marker expression was analyzed 4 days later by flow cytometry. (0.60 MB TIF) [file ppat.1000059.s003.tif]

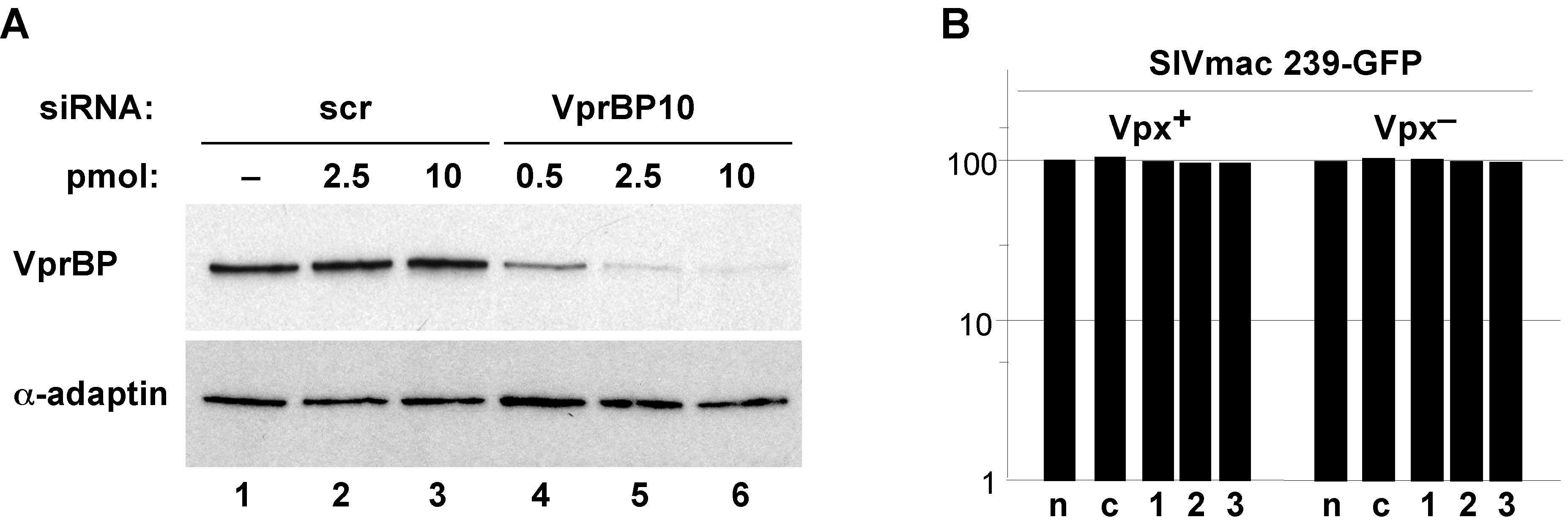

Supplement: Figure S4 — VprBP/DCAF1 is not important for the ability of SIVmac 239 to transduce U2OS cells. RNAi to VprBP was performed in U2OS cells with 0.5, 2.5 and 10 pmol of VprBP10 siRNA, or 2.5 and 10 pmol of nontargeting control siRNA/well in 12 well plates. 2 days after initiation of RNAi cells were (A) harvested for immunoblot analysis of VprBP expression levels, or (B) infected with VSV-G pseudotyped SIVmac 239(GFP) reporter viruses possessing wild type Vpx (Vpx+), or not (Vpx−). GFP-positive cells was quantified by flow cytometry two days later. Transduction efficiencies were normalized to those seen with control U2OS cells that were not subjected to RNAi. Labels at the bottom of the histogram indicate data from U2OS cell populations that have not been subjected to RNAi (n), were treated with 10 pmol of nontargeting siRNA (c), or with 0.5 (1), 2.5 (2) and 10 (3) pmol of VprBP10 siRNA/well in 12 well plates. (0.45 MB TIF) [file ppat.1000059.s004.tif]
